# ARE YOU READY FOR CHANGE? FACTORS ASSOCIATED WITH COMMITMENT TO CHANGE IN CLINICAL PRACTICE

**DOI:** 10.1093/geroni/igz038.005

**Published:** 2019-11-08

**Authors:** Jasleen K Chahal, Kimberly Northrip, Dena Silva

**Affiliations:** 1 UK HealthCare CECentral, Lexington, Kentucky, United States; 2 Incedo - University of North Texas Health Science Center, Fort Worth, Texas, United States

## Abstract

Commitment to Change (CTC) has been shown to be an effective performance measure of continuing professional development (CPD) training, in that 50-67% of providers choosing CTC also made behavioral changes in clinical practice (Domino et al. 2011; Perkins et al., 2007). However, very little is known about the factors that are associated with a learner committing to practice change. This study presents findings from a retrospective observational study that compared trends across various health profession, demographics, and activity-type categories to identify learner characteristics that may lead to higher CTC responses. Learner data from 2014 -2017 was obtained from two different continuing education office databases. The combined dataset contained 68,365 evaluations from 26,508 learners, of which 22.8% wrote a CTC. At both sites, CTC was more likely if the activity was enduring or charged a fee and less likely for an RSS (p<0.001). Results indicate that CTC varies based on provider demographics, such as profession and specialty. Allied health providers (32.4-37.5%) and health educators (36%) were most likely to make a commitment, followed by nurses (28.6%), physicians (23.9%), pharmacists (14.9%), and dentists (9.1%). Physician specialties and provider region also varied with the highest CTC by geriatricians (50%) and occupational medicine providers (42.3%) and the lowest rates of CTC found in the southeast region. Results of the study will help inform the development and implementation of future CPD in order to successfully engage learners in committing to change clinical practices.

